# Body Image During Pregnancy in the Era of Coronavirus Disease 2019: The Role of Heterogeneous Patterns of Perceived Social Support

**DOI:** 10.3389/fpsyg.2021.742525

**Published:** 2021-10-12

**Authors:** Małgorzata Piȩta, Marcin Rzeszutek, Michał Lendzion, Monika Grymowicz, Wojciech Piȩta, Agata Kasperowicz, Marek Kucharski, Mateusz Przybył, Roman Smolarczyk

**Affiliations:** ^1^University of Warsaw, Warsaw, Poland; ^2^Faculty of Psychology, University of Warsaw, Warsaw, Poland; ^3^Medical University of Warsaw, Warsaw, Poland

**Keywords:** pregnancy, body image, social support, COVID-19, person-centered approach

## Abstract

**Objective:** The aim of this study was to explore the profiles of pregnant women on perceived social support with regard to sociodemographic variables, coronavirus disease 2019 (COVID-19)-related distress issues, and body image. We compared the aforementioned relationships within the study variables between pregnant women and a control group of non-pregnant women.

**Method:** The study sample comprised 345 women, 157 pregnant women, and 188 women in the control group. Participants filled out paper-and-pencil or online psychometric questionnaires to assess the variables analyzed in our research.

**Results:** Latent profile analysis revealed six profiles of pregnant women based on perceived social support, which varied in terms of body image evaluation. The high-support profile differed from the profiles with the lowest scores in all support domains. Significant differences in body image between the profiles of pregnant women and the control group were noted.

**Conclusion:** Understanding the mechanisms through which women can attain more body satisfaction during pregnancy is an important research topic that can inspire planning for more effective psychological help, especially in the context of the COVID-19 pandemic and related psychological distress.

## Introduction

Body image is a multidimensional construct in which various thoughts, beliefs, emotions, and behaviors play a dynamic role in the subjective evaluation of the physical appearance of the self and general approach to the own body of an individual (Cash, 2011). Pregnancy is characterized by the experience of important physical changes and significant weight gain that can lead to a sense of loss of control and dissatisfaction with the own body of an individual, which poses a risk of psychological distress among pregnant women, including depression, anxiety, and low self-esteem (Watson et al., 2017). The most common explanation for the relationship between poor body image and pregnant-related distress refers to socio-cultural factors and thinness ideals, which are impossible to maintain during pregnancy (Grogan, 2007; Młozniak and Schier, 2016; Watson et al., 2017). Other theoretical explanations highlight the issue of body image standards among women during pregnancy (Fuller-Tyszkiewicz et al., 2013). Interestingly, authors representing this latter standpoint have provided mixed findings on body image concerns over the course of pregnancy. Although the majority of studies observed an intuitively obvious trend pointing to more negative body image and a drop in body satisfaction (Skouteris et al., 2005; Clark et al., 2009), there are also studies providing evidence for stable or even improved body image during pregnancy (Duncombe et al., 2008; Loth et al., 2011). The unexpected latter result may be linked to the fact that pregnancy is a time when weight gain is not so stigmatizing and also when the reproductive role is more important than physical attractiveness.

Some authors have underlined the role of individual differences in perceived social support, mostly from intimate relationships, in coping with the challenges of this transgressive period (Hodgkinson et al., 2014). Social support is shown to act as a protective factor against body image disturbances and pregnant-related mental disorders (Westdahl et al., 2007; Rashid and Mohd, 2017) and, importantly, may enhance subjective well-being and self-efficacy in coping with stress and anxiety (Ginja et al., 2018). In this study, we focused on the association between perceived support from significant others and body image during pregnancy at the time of the coronavirus disease 2019 (COVID-19) pandemic, as compared with non-pregnant women.

The COVID-19 pandemic is a major stressor that uniquely affects the well-being of pregnant women worldwide (Moyer et al., 2020; Mortazavi et al., 2021). Current studies show that the influence of the pandemic on pregnancy care in hospitals, fear of infection among close relatives, restrictions for visits in hospital and thus poor social support, and social stressors like income/job loss, act as additional predictors of distress of pregnant women (Nanjundaswamy et al., 2020). The history of psychiatric disorders was associated with elevated depressive and even posttraumatic stress disorder (PTSD) symptoms among pregnant women during “lockdown” (Ravaldi et al., 2020), and social distancing also has the potential to amplify body dissatisfaction and increase the motive for thinness among women (Swami et al., 2021). The COVID-19 pandemic creates an increased risk of psychiatric disorders among women during the perinatal period, which substantially limits the resources required to adapt to the pregnancy period.

Despite evidence for the vulnerability of pregnant women during the COVID-19 pandemic (Capobianco et al., 2020; Ravaldi et al., 2020; Hamzehgardeshi et al., 2021; Mortazavi et al., 2021), some studies have indicated a paradoxical possibility of better well-being and lower depression rates compared with pre-pandemic times (Pariente et al., 2020). It seems that pregnant women constitute a highly heterogeneous population, but the majority of studies disregard this fact, following the variable approach only (Hodgkinson et al., 2014; Watson et al., 2015; Morley-Hewitt and Owen, 2020), which ignores the heterogeneity of participants within the study variables. The use of the person-centered perspective in studying pregnant women is relatively new and thus, scarce in the literature (Talmon et al., 2020; Raspovic et al., 2021). This methodological design can help us better understand individual differences in the functioning of pregnant women during the COVID-19 pandemic by extracting different profiles of these women with unique relationships within the analyzed variables.

## Current Study

The aim of this study was twofold. First, we wanted to verify whether we could observe different profiles of pregnant women with regard to perceived social support. Second, we aimed to investigate whether profiles of pregnant women differ regarding sociodemographic variables, COVID-19-related distress issues, such as the subjective rating of COVID-19-related mental difficulties and medical history of depression, and body image. Finally, we aimed to interpret the aforementioned differences in the context of analog values acquired from the control group of non-pregnant women. In other words, we wanted to verify whether being pregnant in the COVID-19 pandemic significantly altered body image and what the role of social support is in this aspect.

To the best of our knowledge, there are no studies conducted with pregnant women that would be useful as a direct source of research hypotheses in the case of this special study design, particularly with a control group of non-pregnant women. We mainly employed an exploratory approach in this study. Based on existing studies within different methodological frameworks (Talmon et al., 2020; Raspovic et al., 2021), we expected that our sample of pregnant women would be heterogeneous in terms of perceived social support and that support would differ in relation to sociodemographic variables, COVID-19-related distress issues, and body image. Finally, we expected that women after childbirth would experience, on average, a more negative body image than those from the comparison group. These relationships may also change if we take into account distinct profiles of perceived support during pregnancy.

## Methods

### Participants

The study sample consisted of 345 women, 157 pregnant women, and 188 non-pregnant controls. The pregnant women were recruited from Princess Anna Mazowiecka Clinical Hospital, Poland. The control group was recruited *via* social media platforms *via* an advertisement prepared by the research group. The study participants filled out paper-and-pencil or online questionnaires and voluntarily participated in this study, with no remuneration provided. In cases of pregnancy, the eligibility criteria included being in the third trimester of pregnancy and being admitted to the hospital for childbirth, which was screened by medical doctors working in the hospitals where the research was conducted. The exclusion criteria included cognitive impairment or major medical complications associated with childbirth, such as possible premature childbirth as diagnosed by doctors.

For the control sample, the inclusion criteria included not being pregnant at the time of conducting this study. The study took place between November and May 2021, a time described as the “second” and “third waves” of the COVID-19 pandemic in Poland^1^. The research project was approved by the Ethics Committee.

### Measures

*Multidimensional Body-Self Relations Questionnaire* (MBSRQ; Cash, 2000). The MBSRQ consists of 69 items making up 10 scales that relate to different areas of body image as follows: the appearance evaluation, the appearance orientation, the fitness evaluation, the fitness orientation, the health evaluation, the health orientation, the illness orientation, the body area satisfaction, the overweight preoccupation, and the self-classified weight. Higher results obtained in all scales except the overweight preoccupation and the self-classified weight scale mean a more favorable assessment of body image. An exploratory factor analysis revealed that the Polish MBSRQ items significantly loaded with the main factors of the scale. Internal consistencies of the subscales were satisfactory (Brytek-Matera and Rogoza, 2015).

*Multidimensional Scale of Perceived Social Support* (MSPSS; Zimet et al., 1988). The MSPSS is a 12-item scale measuring perceived social support from three sources, namely, family, friends, and significant others, with no established population norms. Higher results obtained in the scales mean a more favorable assessment of social support from family, friends, a significant other, or a total assessment of perceived social support. The structure of polish adaptation of the scale is the same as to the original one—exploratory and confirmatory analyses have validated the three-factor structure and confirmed its satisfactory psychometric properties (Adamczyk, 2013).

*COVID-19-related distress*: In this study, we utilized short, but reliable operationalization of the COVID-19 distress based on some other studies published at that time, when we started our research (Gambin et al., 2020; Dragan et al., 2021). More specifically, we asked participants on a Likert 1–5 point scale how stressful (in general) was for them this pandemic and their life during it. The answers varied between 1 (“not at all”) and 5 (“very much”).

### Data Analysis

The data analysis consisted of four consecutive stages. First, descriptive analysis was performed. Mean values, SDs, minimum and maximum values, and values of measures of skewness and kurtosis were computed. Frequencies and percentages were calculated for categorical variables. Second, different profiles of perceived support in the group of pregnant women were extracted using the latent profile analysis (LPA; Vermunt, 2017). LPA is a statistical technique, which enables the exploration of unobserved heterogeneity within a study sample (Lubke and Neale, 2006; Nylund et al., 2007). In other words, this method allowed us to classify pregnant women into several exclusive subgroups, characterized by different profiles of perceived social support. A model with an optimal number of such profiles is selected based on several indicators. In this study, we based on the following indicators: Akaike information criterion (AIC), approximate weight of evidence (AWE), Bayesian information criterion (BIC), classification likelihood criterion (CLC), and Kullback information criterion (KIC; Lubke and Neale, 2006). Third, the sociodemographic variables and body image were compared within the subgroups of pregnant women with different support profiles with the use of Pearson's chi-squared test for independence and ANOVA followed by the Gabriel *post-hoc* test. Fourth, the differences detected were then interpreted in the context of the values acquired in the control group, which were used as a reference with the use of the planned contrast test. Conventional cut-off value *p* < 0.05 was used. Calculations were performed with the use of the tidy LPA package (Rosenberg et al., 2018) working in the R Statistics 4.1.0 environment and IBM SPSS Statistics 27.0, Chicago, IL.

## Results

Table 1 presents the sociodemographic characteristics of all the study participants, with statistical tests for differences between the groups.

**Table 1:** Sociodemographic variables in the two study samples of women (*N* = 345).

**Variable**	**Control**	**Pregnant**	
	***N* (188)**	***N* (157)**	
Age in years (M ± SD)	31.05 ± 8.57	31.94 ± 4.60	*t*_(296.17)_ = −1.22, *p* > 0.05
Relationship status			$\chi_{\left(1\right)}^{2}$ = 30.52, *p* < 0.001
Stable relationship	147 (79.5%)	155 (98.7%)	
Single	38 (20.5%)	2 (1.3%)	
Education			$\chi_{\left(3\right)}^{2}$ = 3.52, *p* > 0.05
Elementary	2 (1.1%)	5 (3.2%)	
Vocational	1 (0.5%)	3 (1.9%)	
Secondary	54 (28.7%)	41 (26.1%)	
Higher education	131 (69.7%)	108 (68.8%)	
Employment			
Full employment	151 (80.3%)	126 (80.3%)	$\chi_{\left(2\right)}^{2}$ = 9.33, *p* < 0.01
Unemployed	36 (19.1%)	22 (14.0%)	
Other	1 (0.5%)	9 (5.7%)	
Place of residence			$\chi_{\left(4\right)}^{2}$ = 20.52, *p* < 0.001
Up to 20.000 inhabitants	24 (12.8%)	39 (24.8%)	
21.000-100.000 inhabitants	22 (11.7%)	33 (21.0%)	
101.000-500.000 inhabitants	15 (8.0%)	16 (10.2%)	
More than 500.000 inhabitants	126 (67.0%)	68 (43.3%)	
No permanent residence	1 (0.5%)	1 (0.6%)	
Financial situation			$\chi_{\left(4\right)}^{2}$ = 37.46, *p* < 0.001
Very good	28 (14.9%)	13 (8.3%)	
Good	62 (33.0%)	96 (61.1%)	
Average	78 (41.5%)	48 (30.6%)	
Bad	17 (9.0%)	0 (0%)	
Very bad	3 (1.6%)	0 (0%)	
Quarantine or home isolation			$\chi_{\left(2\right)}^{2}$ = 13.29, *p* < 0.01
Yes	59 (31.4%)	37 (23.6%)	
Limited going outside	54 (28.7%)	27 (17.2%)	
No	75 (39.9%)	93 (59.2%)	
Partner in quarantine or home isolation			$\chi_{\left(2\right)}^{2}$ = 1.16, *p* > 0.05
Yes	21 (11.2%)	21 (13.4%)	
No	162 (86.2%)	134 (85.4%)	
Other	5 (2.7%)	2 (1.3%)	
Diagnosed with SARS-CoV-2			$\chi_{\left(2\right)}^{2}$ = 4.40, *p* > 0.05
Participants	20 (10.6%)	17 (10.8%)	
Participants' partners	18 (9.6%)	6 (3.8%)	
None	150 (79.8%)	134 (85.4%)	
Epidemic situation affected financial status			$\chi_{\left(2\right)}^{2}$ = 2.47, *p* > 0.05
Yes	37 (19.7%)	22 (14.0%)	
To a slight degree	60 (31.9%)	48 (30.6%)	
No	91 (48.4%)	87 (55.4%)	
Experiencing mental difficulties due to epidemic situation			$\chi_{\left(2\right)}^{2}$ = 31.91, *p* < 0.001
Yes	61 (32.4%)	32 (20.4%)	
To a slight degree	85 (45.2%)	44 (28.0%)	
No	42 (22.3%)	81 (51.6%)	
Having children	132 (70.2%)	90 (57.3%)	$\chi_{\left(1\right)}^{2}$ = 6.19, *p* < 0.05
Diagnosed with depression or other disorders	44 (23.4%)	4 (2.5%)	$\chi_{\left(1\right)}^{2}$ = 31.07, *p* < 0.001
A loved one diagnosed with depression or other disorders	117 (62.2%)	22 (14.0%)	$\chi_{\left(1\right)}^{2}$ = 82.69, *p* < 0.001

*M, mean; SD, standard deviation; t, independent sample t-test; χ^2^ chi-squared test for independence*.

In the group of pregnant women, more women were in stable relationships, fewer women were unemployed, and fewer women lived in a city with more than 500,000 inhabitants. Their financial situation was significantly better, fewer women were in quarantine, fewer women experienced mental difficulties due to epidemic situations, fewer women already had children, fewer women were diagnosed with mental disorders themselves, and fewer women knew that someone close was diagnosed with a mental disorder. Out of 157 pregnant women, 65 women (41.4%) planned to give birth by cesarean section, and 78 women (49.7%) planned natural childbirth.

Table 2 presents the descriptive statistics for the analyzed variables.

**Table 2:** Descriptive statistics for variables in the study sample (*N* = 345).

	***M***	***SD***	**Min**	**Max**	***S***	***K***
**Support**						
Significant other	6.03	1.33	1.00	7.00	−1.82	3.22
Family	5.30	1.51	1.00	7.00	−0.86	0.09
Friends	5.48	1.33	1.00	7.00	−1.08	1.07
Total	5.60	1.21	1.00	7.00	−1.36	2.22
**Body image**						
Appearance evaluation	3.45	0.90	1.00	5.00	−0.60	−0.23
Appearance orientation	3.14	0.57	1.75	4.67	0.18	−0.08
Fitness evaluation	3.14	0.94	1.00	5.00	−0.30	−0.50
Fitness orientation	3.08	0.85	1.00	5.00	0.00	−0.52
Health evaluation	3.65	0.73	1.00	5.00	−0.52	0.21
Health orientation	3.26	0.59	1.50	4.63	−0.24	−0.12
Illness orientation	3.24	0.77	1.20	5.00	−0.05	−0.44
Overweight preoccupation	2.52	0.89	1.00	5.00	0.38	−0.49
Body areas satisfaction scale	3.31	0.77	1.00	5.00	−0.53	0.12
Self-classified weight	3.19	0.73	0.50	5.00	−0.31	1.68

*M, mean; SD, standard deviation; min, minimal value; max, maximum value; S, skewness; K, kurtosis*.

Distributions of support from significant others, friends, and total support were leptokurtic and negatively skewed. Self-classified weight in body image was also leptokurtic. Other variables did not differ from normal distributions in terms of range or symmetry.

The scores reflecting support from significant others, family, and friends were submitted to LPA. The analysis was performed on a group of pregnant women.

**Figure 1:**
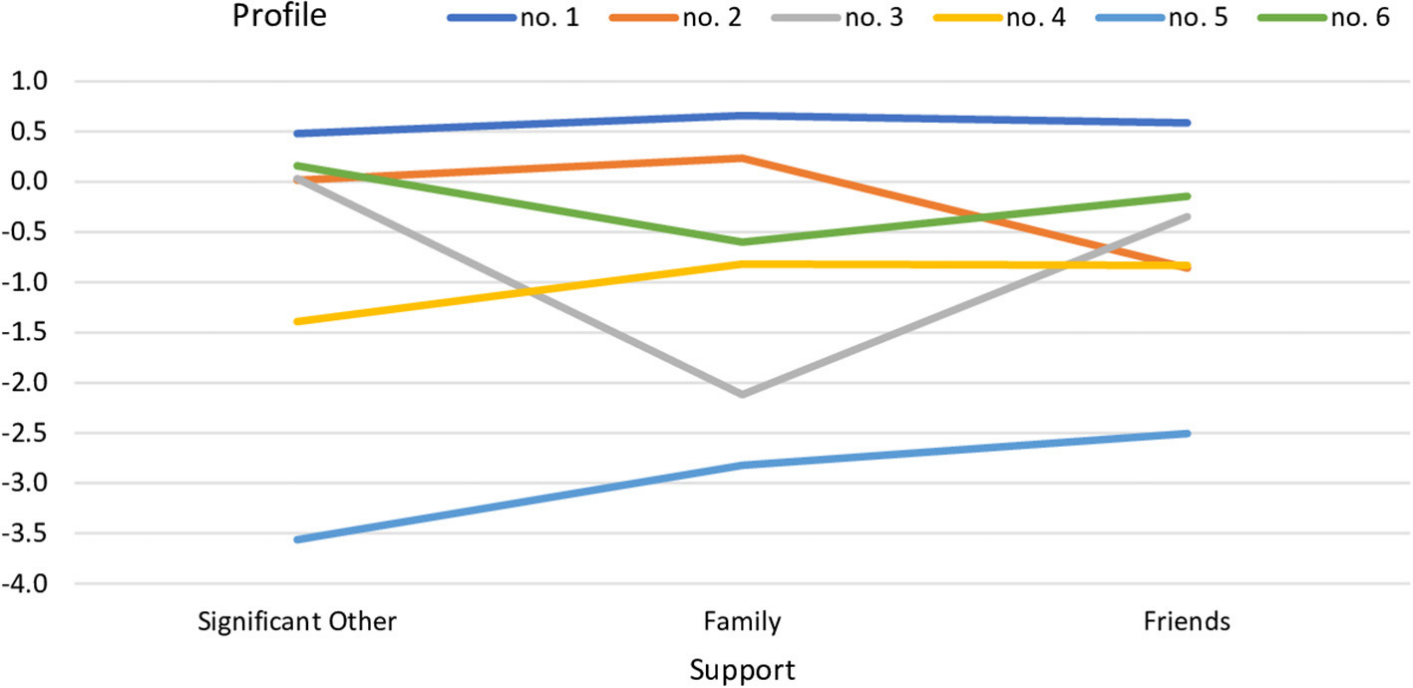
Profiles of perceived support in the group of pregnant women.

An analytic hierarchy process, based on the fit indices, i.e., AIC, AWE, BIC, CLC, and KIC (Akogul and Erisoglu, 2017), suggested the best-fitted model in the form of the six profile solution. The best-fitted model was accepted based on the lowest values of the aforementioned fit indices (see Figure 1). Values of all fit indices for six solutions are provided in Table 3.

**Table 3:** Values of fit indices for solutions with different numbers of profiles.

**No. of profiles**	**AIC**	**AWE**	**BIC**	**CLC**	**KIC**
1	1,345.63	1,410.31	1,363.97	1,335.63	1,354.63
2	1,116.82	1,225.96	1,147.38	1,098.80	1,129.82
3	994.26	1,147.95	1,037.05	968.14	1,011.26
4	948.44	1,146.59	1,003.45	914.32	969.44
5	901.02	1,143.60	968.26	858.91	926.02
**6**	**889.44**	**1,176.54**	**968.90**	**839.27**	**918.44**

*AIC, Akaike information criterion; AWE, approximate weight of evidence; BIC, Bayesian information criterion; CLC, classification likelihood criterion; KIC, Kullback information criterion*.

*The values of fit indices for the retained profile solution are in bold*.

The first profile (*n* = 88) was characterized by the highest level of support from all three sources, the second profile (*n* = 16) was characterized by an average level of support with a lower level of support from friends, and the third profile (*n* = 6) was characterized by a low level of family support. The fourth profile (*n* = 15) was characterized by a low level of support from significant others, the fifth profile (*n* = 7) was characterized by a low level of support from all three sources, and the sixth profile (*n* = 25) was characterized by an average level of support with lower support from family.

In the next stage of analysis, participants from the extracted profiles were compared in terms of socioeconomic data. As seen in Table 4, the number of participants in stable relationships was significantly lower in profile 5 (low level of support from all three sources) and profile 6 (average level of support with lower support from family) groups, $\chi_{\left(5\right)}^{2}$ = 12.51, *p* < 0.05.

**Table 4:** Number of participants in stable relationships depending on the profile of perceived support.

**Profile**	***n* (%)**
No. 1	88 (100%)
No. 2	16 (100%)
No. 3	6 (100%)
No. 4	15 (100%)
No. 5	6 (85.7%)
No. 6	24 (96.0%)

In the profile 3 group (low level of family support), both the number of participants diagnosed with mental disorders and those in a relationship with someone diagnosed with a mental disorder were higher than in groups of participants with other profiles of perceived support (Table 5).

**Table 5:** Number of participants diagnosed with mental disorders or in a relationship with someone diagnosed with a mental disorder depending on the profile of perceived support.

**Profile**	**Participant**	**Someone close**
	***n* (%)**	***n* (%)**
No. 1	1 (1.1%)	11 (12.5%)
No. 2	1 (6.3%)	1 (6.3%)
No. 3	2 (33.3%)	4 (66.7%)
No. 4	0 (0%)	2 (13.3%)
No. 5	0 (0%)	0 (0%)
No. 6	0 (0%)	4 (16.0%)

There were no statistically significant relationships between profiles of perceived support and education, $\chi_{\left(15\right)}^{2}$ = 22.59, *p* > 0.05, employment, $\chi_{\left(15\right)}^{2}$ = 13.02, *p* > 0.05, place of residence, $\chi_{\left(20\right)}^{2}$ = 11.98, *p* > 0.05, financial situation, $\chi_{\left(10\right)}^{2}$ = 9.61, *p* > 0.05, being in quarantine, $\chi_{\left(10\right)}^{2}$ = 8.18, *p* > 0.05, being in relationship with a partner in quarantine, $\chi_{\left(10\right)}^{2}$ = 3.09, *p* > 0.05, being diagnosed with severe acute respiratory syndrome coronavirus 2 (SARS-CoV-2), $\chi_{\left(10\right)}^{2}$ = 11.64, *p* > 0.05, the impact of the pandemic on financial situation, $\chi_{\left(10\right)}^{2}$ = 17.46, *p* > 0.05, experiencing mental difficulties due to the pandemic, $\chi_{\left(10\right)}^{2}$ = 10.28, *p* > 0.05, and already having children, $\chi_{\left(5\right)}^{2}$ = 7.15, *p* > 0.05.

The extracted groups with different profiles of support were also compared in terms of body image. Table 6 presents the mean values of body image indicators with a one-way ANOVA.

**Table 6:** Mean values of body image indicators with values of one-way ANOVA.

	**Profile 1**		**Profile 2**		**Profile 3**		**Profile 4**		**Profile 5**		**Profile 6**		***F***	**df**	***p***	**η^**2**^**
	***M***	***SD***	***M***	***SD***	***M***	***SD***	***M***	***SD***	***M***	***SD***	***M***	***SD***				
Appearance evaluation	3.63	0.76	3.43	0.76	3.43	0.91	3.09	0.71	2.78	0.66	3.50	0.83	2.62	5.151	0.026	0.08
Appearance orientation	3.18	0.52	3.18	0.52	3.28	0.52	2.99	0.41	3.07	0.38	2.97	0.59	1.05	5.151	0.392	0.03
Fitness evaluation	3.18	0.89	3.31	0.43	3.11	1.13	2.87	0.71	2.71	0.71	3.29	0.87	1.00	5.151	0.419	0.03
Fitness orientation	3.16	0.75	3.13	0.50	2.96	0.66	2.65	0.38	2.69	0.41	2.83	0.76	2.37	5.151	0.042	0.07
Health evaluation	3.85	0.59	3.49	0.67	3.53	0.92	3.31	0.59	3.67	0.72	3.72	0.59	2.64	5.151	0.026	0.08
Health orientation	3.39	0.55	3.31	0.59	3.67	0.67	3.03	0.42	3.16	0.47	3.17	0.64	2.03	5.151	0.078	0.06
Illness orientation	3.38	0.72	3.30	0.71	3.13	0.95	3.09	0.70	3.03	0.99	2.97	0.82	1.48	5.151	0.201	0.05
Overweight preoccupation	2.44	0.70	2.52	1.15	2.71	1.18	2.47	0.72	2.18	0.95	2.21	0.98	0.63	5.151	0.673	0.02
Body areas satisfaction scale	3.38	0.78	3.26	0.47	3.28	0.94	2.91	0.77	2.78	0.85	3.38	0.68	1.77	5.151	0.123	0.06
Self-classified weight	3.23	0.71	3.13	0.76	3.42	0.97	2.87	1.36	3.43	0.93	3.04	0.80	0.88	5.151	0.495	0.03

*M, mean; SD, standard deviation; F, one-way ANOVA; df, degrees of freedom; p, statistical significance; η^2^, partial eta-squared measure of effect size*.

**Figure 2:**
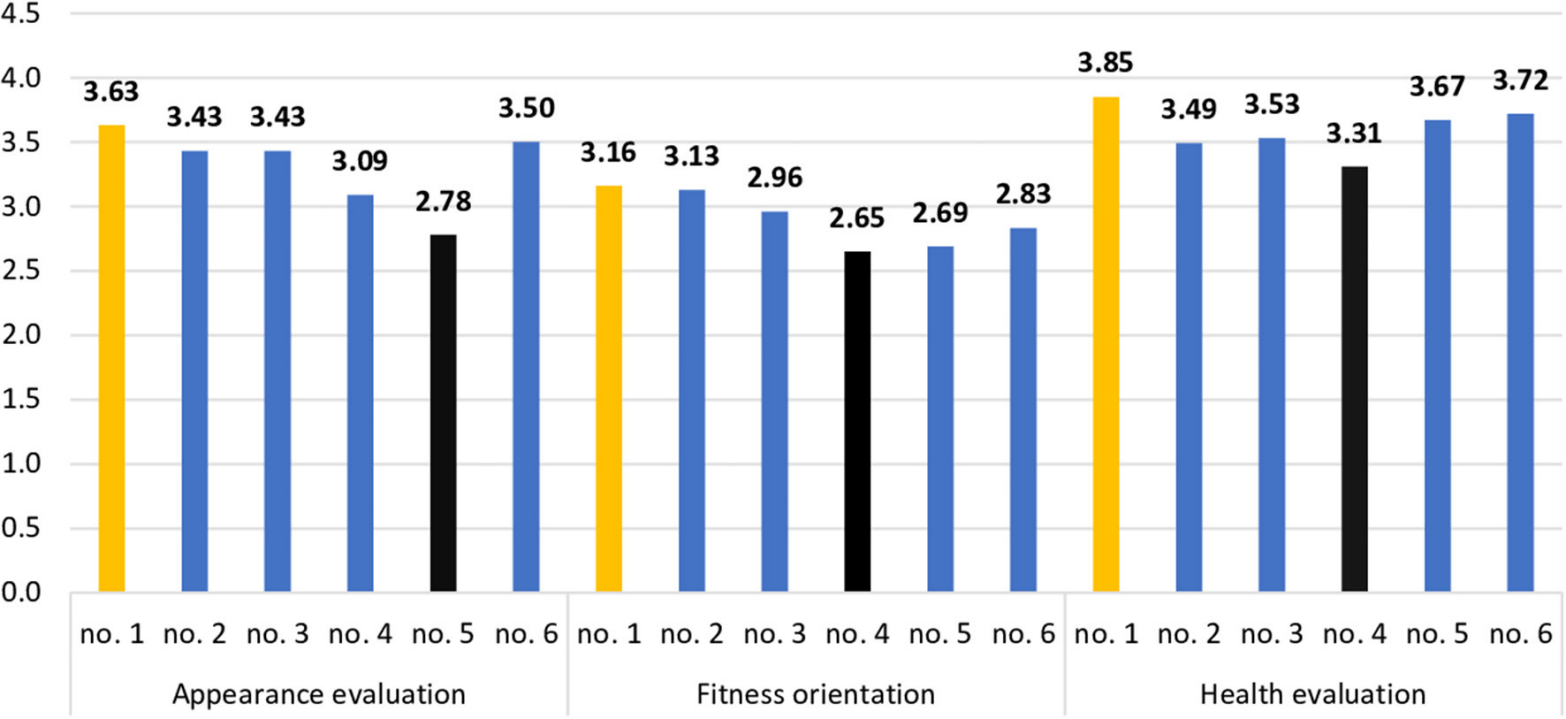
Statistically significant relationships between profiles of perceived support and body image in the group of pregnant women.

There were statistically significant differences between the extracted profiles regarding appearance evaluation, fitness orientation, and health evaluation (see Figure 2). According to the values of Gabriel, *post-hoc* test profile 1 (highest level of support) differed significantly from profile 5 (low level of support) in terms of appearance evaluation, *t* = 2.82, *p* < 0.05, and from profile 4 (low level of support from significant other) in terms of fitness orientation, *t* = 2.65, *p* = 0.068, and health evaluation, *t* = 3.11, *p* < 0.05.

In the final analysis, the groups of pregnant women characterized by profile 1 and profiles 4 and 5 combined, i.e., groups that differed significantly in terms of body image, were compared to the control group. For this analysis, groups with profiles 4 and 5 were combined into a single group of participants with lower social support. The values from the control group were used as a reference. Table 7 presents the mean values of body image indicators acquired in the three groups compared with the values of one-way ANOVA.

**Table 7:** Mean values of body image indicators with one-way ANOVA.

	**Control group**		**Profile 1**		**Profile 4 or 5**		***F***	**df**	***p***	**η^**2**^**
	***M***	***SD***	***M***	***SD***	***M***	***SD***				
Appearance evaluation	3.41	0.97	3.63	0.76	2.99	0.70	4.80	2.295	0.009	0.03
Fitness orientation	3.12	0.96	3.16	0.75	2.66	0.38	3.01	2.295	0.051	0.02
Health evaluation	3.59	0.81	3.85	0.59	3.42	0.64	4.78	2.295	0.009	0.03

*M, mean; SD, standard deviation; F, one-way ANOVA; df, degrees of freedom; p, statistical significance; η^2^, partial eta-squared measure of effect size*.

There were statistically significant differences regarding appearance evaluation and health evaluation and differences close to statistical significance regarding fitness orientation. For the purpose of comparison with the control group, contrast tests were used. Regarding appearance evaluation, there was a difference close to statistical significance between the control group and participants with profile 1, *t* = 1.85, *p* = 0.065, and a statistically significant difference between the control group and participants with profiles 4 or 5, *t* = −2.10, *p* < 0.05. Regarding fitness orientation, there was a statistically significant difference between the control group and participants with profile 4 or 5, *t* = −2.31, *p* < 0.05, but there was no difference between the control group and participants with profile 1, *t* = 0.38, *p* > 0.05.

**Figure 3:**
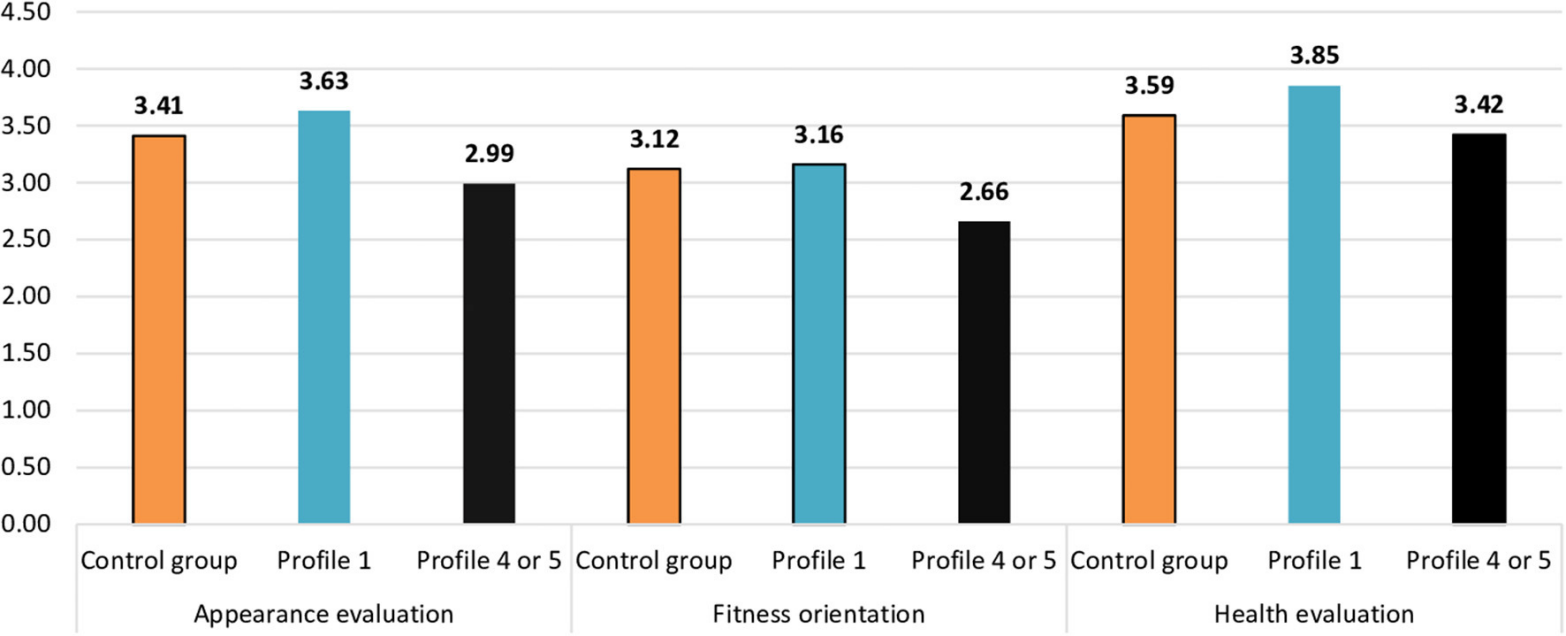
Statistically significant relationships between profiles of perceived support and body image between pregnant women and the control group.

Regarding health evaluation, there was a statistically significant difference between the control group and participants with profile 1, *t* = 2.70, *p* < 0.01, but there was no difference between the control group and participants with profile 4 or 5, *t* = −1.00, *p* > 0.05 (see Figure 3). The appearance evaluation in the group of pregnant women with profile 1 (highest level of support) was higher than in the control group. The appearance evaluation in the group of pregnant women with profiles 4 or 5 (low level of support from significant others or in general) was lower than in the control group. Fitness orientation in the group of pregnant women with profile 4 or 5 (low level of support from significant other or in general) was lower than in the control group. Fitness orientation in the group of pregnant women with profile 1 (highest level of support) was similar to fitness orientation in the control group. Health evaluation in the group of pregnant women with profile 1 (highest level of support) was higher than in the control group. Health evaluation in the group of pregnant women with profile 4 or 5 (low level of support from significant other or in general) was similar to the control group.

## Discussion

The results of this study were mostly in accordance with our explorative research hypotheses. First, we observed six profiles of pregnant women based on perceived social support, which varied in terms of body image evaluation. This study is the first to explain heterogeneity in the approach to the own body of an individual during pregnancy, depending on different social support profiles. More specifically, the largest group of women extracted in this study was characterized by high levels of support provided by significant others, as well as family and friends, and was associated with more positive self-evaluation in distinct body image subscales.

Pregnant women from the high-support profile differed significantly from the pregnant women from the profile with the lowest scores in all support domains in the context of appearance evaluation. Also, women with the highest perceived support levels varied from women characterized by low levels of support from significant others in terms of fitness orientation and health evaluation.

Pregnant women who perceive more social support in their close relationships tend to assess their changing bodies in more positive ways throughout pregnancy. A positive perception of the body during pregnancy allows women to maintain their feeling of being socially attractive, despite objective changes in their bodies (Hodgkinson et al., 2014; Ginja et al., 2018). This is in line with the argument of Schier (2021) that body image is a dynamic construct that can be influenced by actions and physical changes associated with the body and its role in specific life circumstances, especially if they are formative for the own identity of an individual.

In addition, the observed profiles of pregnant women with perceived support were also associated with some of the sociodemographic and medical variables controlled in this study. First, we observed fewer participants in stable relationships among pregnant women belonging to profiles characterized by low levels of perceived support from all sources. Also, participants with a low level of family support were more often diagnosed with mental disorders or had been in a relationship with someone with psychiatric illness in the past. The co-occurrence of mental health problems and low levels of social support has important clinical and social consequences, as social support is shown to act as an important protective factor in mental health maintenance (Westdahl et al., 2007; Rashid and Mohd, 2017). In particular, mental health problems during pregnancy can have important consequences for both maternal and fetal outcomes and are linked to an increased risk of postpartum depression.

Interestingly, no other sociodemographic variables or COVID-19-related variables were significantly associated with the profiles of participants based on levels of perceived social support. This result is in line with studies conducted among women delivering during the first “lockdown,” as they were found to be exposed to lower postpartum depression risk than women delivering before the COVID-19 pandemic (Pariente et al., 2020). We also found that pregnant women reported experiencing fewer mental difficulties due to the pandemic and were less often forced to undergo quarantine than women in the control group. Accordingly, women during pregnancy experienced less negative financial consequences of the pandemic compared to the control group.

The aforementioned surprising results, particularly the null result with COVID-19 distress, can be explained by the greater support that future mothers have in their situation of pregnancy compared to non-pregnant women who often cannot expect such support (Pariente et al., 2020). Furthermore, for pregnant women, having a partner plays a particularly important role in their psychological resilience (Harville et al., 2009; Khatri et al., 2018; Pariente et al., 2020). It may also be that pregnant women, who are more emotionally engaged in their close environment, such as circumstances external to their household and family life, were impacted by COVID-19-related distress to a lesser degree.

Finally, we noticed differences in body image between pregnant women and women in the control group. Pregnant women from profiles with very high levels of perceived social support evaluated their bodies (i.e., appearance evaluation, health evaluation, and fitness orientation) much more positively than women from the control group. In other words, not all pregnant women assessed their bodies more favorably than women who were not pregnant—it depended largely on the level of perceived support. In most body image domains, pregnant women did not report significant differences when compared to the control group. Only profiles of pregnant women with the highest and the lowest levels of perceived social support differed from the control group in terms of appearance, health evaluation, and fitness orientation. In addition, alternative explanations of the obtained results cannot be overlooked and should also be considered in further research. In particular, further studies can explore the possible influence of some other latent factors, such as personality traits, that can influence both the body image attitudes and perceived social support, even if the two variables are not correlated.

It seems that pregnant women are a very heterogeneous group, which can be easily overlooked in studies exclusively following the variable-centered approach (Hodgkinson et al., 2014; Watson et al., 2015). In other words, it is impossible to draw universal conclusions on body image in pregnancy in general, but rather, the focus should be on specific profiles of pregnant women with regard to psychosocial factors uniquely related to particular body image profiles.

## Strengths and Limitations

Our study has several strengths, including the comparison of pregnant women with a control group of non-pregnant women and the person-centered approach, which adds value to the literature on body image during pregnancy (Morley-Hewitt and Owen, 2020; Raspovic et al., 2021). Nevertheless, our study was not free from limitations. First, the study design was cross-sectional, so no cause-and-effect relationships could be drawn from its results. Second, the study sample was not very large and limited to women delivering in one hospital, which makes it difficult to draw representative conclusions of study findings for overall populations of pregnant women during the COVID-19 pandemic. Finally, we did not gather extensive information about the medical history and medical variables of the women.

## Conclusion

Despite these limitations, this study offers some new insights into the psychological situation of pregnant women in the global pandemic. The possibility of gaining more body satisfaction during pregnancy is an important research perspective that can shed new light on the processes underlying potential identity change triggered by motherhood (Hodgkinson et al., 2014; Ginja et al., 2018). This study can be used for more efficient planning of potential psychological help for women in the perinatal period based on a better understanding of their heterogeneity and specific needs. In the future, longitudinal studies could be conducted that enable the discovery of causal relationships between study variables. Also, the psychological distress associated with the COVID-19 pandemic should be further explored, as the global health crisis can be a reason for long-term consequences that are not captured by current research.

## Data Availability Statement

The raw data supporting the conclusions of this article will be made available by the authors, without undue reservation.

## Ethics Statement

The studies involving human participants were reviewed and approved by Ethics Committee, Faculty of Psychology, University of Warsaw. Written informed consent for participation was not required for this study in accordance with the national legislation and the institutional requirements.

## Author Contributions

All authors listed have made a substantial, direct and intellectual contribution to the work, and approved it for publication.

## Funding

This study was financed by the project Excellence Initiative – Research University funded by the Ministry of Science and Higher Education under the internal grant for the University of Warsaw in cooperation with the Medical University of Warsaw (number: 501-D125-20-0004319).

## Conflict of Interest

The authors declare that the research was conducted in the absence of any commercial or financial relationships that could be construed as a potential conflict of interest.

## Publisher's Note

All claims expressed in this article are solely those of the authors and do not necessarily represent those of their affiliated organizations, or those of the publisher, the editors and the reviewers. Any product that may be evaluated in this article, or claim that may be made by its manufacturer, is not guaranteed or endorsed by the publisher.

## Footnotes

^1^Portal Gov.pl (2021). Retrieved 12 July 2021, from https://www.gov.pl/.
